# Impact of number of channels on RF shimming at 3T

**DOI:** 10.1007/s10334-012-0360-5

**Published:** 2013-01-13

**Authors:** Alexander S. Childs, Shaihan J. Malik, Declan P. O’Regan, Joseph V. Hajnal

**Affiliations:** 1 Division of Imaging Sciences and Biomedical Engineering, King’s College London, The Rayne Institute, 4th Floor Lambeth Wing, St. Thomas’ Hospital, London, SE1 7EH UK; 2 Robert Steiner MRI Unit, Imaging Sciences Department, MRC Clinical Sciences Centre, Imperial College London, Hammersmith Hospital, London, UK

**Keywords:** Parallel Transmission, RF shimming, Image nonuniformity, SAR control

## Abstract

**Object:**

At high-field strengths (≥3T) inhomogeneity of the radio frequency (RF) field and RF power deposition become increasingly problematic. Parallel Transmission (PTx)—the use of segmented transmission arrays with independently driven elements—affords the ability to combat both of these issues. There are a variety of existing designs for PTx coils, ranging from systems with two channels to systems with eight or more. In this work, we have investigated the impact of the number of independent channels on the achievable results for both homogeneity improvement and power reduction in vivo.

**Materials and methods:**

A 3T Philips Achieva MRI system fitted with an 8-channel PTx body coil was driven so as to emulate configurations with 1, 2 4 and 8 independent channels. RF shimming was used in two different anatomies in order to assess improvements in RF homogeneity.

**Results:**

Significant homogeneity improvements were observed when increasing from 1 to 2, 2 to 4, and 4 to 8 channel configurations. Reductions in RF power requirements and local SAR were predicted for increasing numbers of channels.

**Conclusion:**

Increasing the number of RF transmit channels adds extra degrees of freedom which can be used to benefit homogeneity improvement or power reduction for body imaging at 3T.

## Introduction

At high field strengths (3T and greater), the excitation radio-frequency (RF) wavelength becomes comparable to the size of the human body, causing strong coil–tissue interactions which are subject-specific, depending on geometry and tissue electrical properties. The result of these interactions is an inhomogeneous RF transmit (B_1_^+^) field, which leads to spatially varying image contrast. Furthermore, conventional single-channel RF systems generally have a one-time adjustment to match the RF coil to a nominal load presented by an “average patient”, whereas in fact the loading of the RF coil is subject-dependent, which can result in variable performance. The use of multiple transmit channels offers a means for mitigating these effects by allowing for greater control over the RF electromagnetic fields. RF shimming [1], in which a subject-specific adjustment is made to the relative amplitudes and phases of each transmit channel, can be used to improve B_1_^+^ homogeneity and/or reduce SAR.

While such techniques have been technically demonstrated for transmit arrays with eight or more channels [2, 3], systematic clinical studies of RF shimming have primarily used commercially available two channel systems [4]. RF shimming at 3T has been used to stabilise and improve B_1_^+^ homogeneity while achieving shorter image acquisition times compared with single-channel quadrature excitation [4], and to also improve diagnostic quality of resulting images [5]. These improvements come directly from the ability to manipulate the spatial B_1_^+^ distribution as a linear sum of contributing fields from individual coil elements:

1 $$ S({\mathbf{r}}) = \sum\limits_{i = 1}^{Nc} {\sigma_{i} ({\mathbf{r}})b_{i} } $$

In the above expression, *σ*_*i*_(**r**) is the transmit sensitivity of the *i*th coil, N_c_ is the total number of coils, *S*(**r**) is the overall transmit sensitivity, and b_i_ are complex weights applied to each channel. Transmit sensitivity is defined as the ratio of the actual B_1_^+^ field to the nominal value; it is a dimensionless value which describes spatial variation of the field amplitude and phase. Equation 1 shows that the ability to obtain uniform overall sensitivity depends on the sensitivities of the different coils in the array. It might be expected, therefore, that more coils, or perhaps different coil designs, would yield even larger improvements than a two-channel configuration. In this investigation, a 3T MRI scanner with eight physical transmit channels has been used to emulate different 2-, 4- and 8-channel configurations in vivo. Possible benefits of RF shimming were assessed in a multiple-volunteer study focusing on B_1_^+^ homogeneity, image quality and RF power reduction.

## Methods

Experiments were performed using an 3T Achieva MRI system (Philips Healthcare, Best, The Netherlands) equipped with an 8-channel parallel transmit (PTx) body coil. The coil [3] consists of an array of eight elements, arranged around the bore of the magnet to give maximum spatial variation in transmit sensitivity in the axial plane.

### RF shim framework

The overall transmit sensitivity of the array coil is a linear sum depending on the complex weighting applied to each channel (Eq. 1). This can be rewritten in matrix form as **S** = **σb,** where **S** is an *N* × 1 vector (*N* is the number of voxels), **σ** is an *N* × *N*_c_ sensitivity matrix and **b** is an *N*_c_ × 1 vector containing the complex coil weightings for each channel. For the system used, *N*_c_ = 8 and **b** = [1 1 1 1 1 1 1 1]^T^ is defined as quadrature mode (single-channel) transmission. This definition of “quadrature” mode depends on the system being calibrated; this will be discussed later. Emulation of systems with a reduced number of channels can be performed by introducing an *N*_c_ × *N*_r_ channel reduction matrix, **R**. This reduction matrix allows for emulation of systems with *N*_r_ < *N*_c_ channels via linear combinations of the scanner’s physical channels. The transmit sensitivity is then written **S** = **σRb**, and **b** represents the weightings applied now not to individual physical coils, but to the N_r_ emulated channels. Full control of the physical coils implies **R** is the *N*_c_ × *N*_c_ identity matrix.

The phase/amplitude relationship between all channels that defines quadrature mode is a property of the system hardware that must be determined by calibration. For our system, this is done using a standard phantom. Choice of the calibration conditions does not affect full 8-channel operation. However, it adds a bias to reduced channel combinations by locking in some aspects of the calibration. In order to reduce the effect that this has, a new system calibration matrix was computed by performing full 8-channel shimming on 10 previously acquired pelvic B_1_^+^ maps (10 different subjects) individually, then taking the complex average of all results for each channel. The resulting weights *c*_i_,which provide an optimised average starting shim, can be incorporated into a diagonal matrix, **C**_ii_ = *c*_i_ which is included in the overall expression for the combined system sensitivity: **S** = **σCRb**. The calibration matrix was explicitly defined prior to any experimental work and not altered subsequently.

RF Shim weights were calculated using Magnitude Least Squares (MLS) optimisation [6, 7] using the local variable exchange method

2 $$ {\text{b}} = { \hbox{min} }\left\{ { \, \left\| {\left| {\sigma CRb - \, S_{target} } \right|} \right\|^{ 2} + \, \lambda \left\| {\text{Rb}} \right\|^{ 2} } \right\} $$

**S**_**target**_ is the target combined transmit sensitivity; in this work **S**_**target**_ = 1.0 for all space. The regularisation parameter λ is designed to control total RF power. Note that the solution, **b**, has N_r_ entries; the actual weights applied to the N_c_ physical coils are determined from the product **Rb**. Hence the second term in Eq. 2, which enumerates the total RF power, considers this product. The overall scaling of **R** is not important in the term λ||**Rb**||^2^ since scalar factors in **R** can be subsumed into the parameter λ.

Several candidate reduction matrices for emulating 2- and 4-channel systems were tested on the pilot data. Candidates included phased combinations, Fourier modes [2] and pair-wise coil combinations. These were evaluated using Eq. 2 with a range of values of λ to find the combinations that produced the optimal combination of B_1_^+^ homogeneity and lowest total RF power when applied to the pilot data. The optimised 4-channel reduction matrix used in this study is shown below; in this case adjacent coils were paired together, corresponding to creation of an enveloping array with fewer, larger coil elements placed adjacently.

3 $$ {\mathbf{R}}^{T} = \left( {\begin{array}{*{20}c} 1 & 1 & 0 & 0 & 0 & 0 & 0 & 0 \\ 0 & 0 & 1 & 1 & 0 & 0 & 0 & 0 \\ 0 & 0 & 0 & 0 & 1 & 1 & 0 & 0 \\ 0 & 0 & 0 & 0 & 0 & 0 & 1 & 1 \\ \end{array} } \right) . $$

Two 2-channel combinations were used. The first used the following reduction matrix

4 $$ {\mathbf{R}}^{T} = \left( {\begin{array}{*{20}c} 1 & 1 & 0 & 0 & 1 & 1 & 0 & 0 \\ 0 & 0 & 1 & 1 & 0 & 0 & 1 & 1 \\ \end{array} } \right) , $$

which was found to yield the best results in the pilot data. The second was chosen to emulate orthogonal, nominally linear modes of a 2-channel birdcage system and was defined as:

5 $$ {\mathbf{R}}^{T} = \frac{1}{2}\left( {\begin{array}{*{20}c} 2 & {1 - i} & 0 & {1 + i} & 2 & {1 - i} & 0 & {1 + i} \\ 0 & {1 + i} & 2 & {1 - i} & 0 & {1 + i} & 2 & {1 - i} \\ \end{array} } \right). $$

### RF shimming

**Fig. 1:**
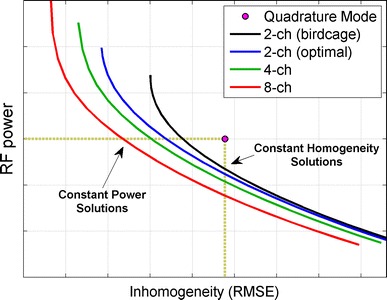
Trade off between relative RF power and inhomogeneity (RMSE) for different coil configurations. Each *curve* is generated by performing the RF shimming calculation for multiple values of regularization parameter λ. Solutions for constant RF power are indicated by the intersection of the horizontal dotted line with the other curves. Solutions for constant homogeneity are indicated by intersection with the *vertical dotted line*

For each subject, experiments were performed using the three different combination matrices described above, as well as 8-channel optimization (**R** = **I**). In each case, error vs. power curves (“L-curves”) were plotted by performing the optimisation (Eq. 2) for multiple values of λ. The MLS algorithm, implemented in MATLAB 2009b (The Mathworks, Natick, MA) on a desktop PC (Windows XP, Dual core CPU 2.4 GHz, 2 GB RAM), calculated all shim settings for a subject in less than 10 s, allowing for straightforward implementation in a scanning session. The curves were used to select two solutions for each case: the first maximised the homogeneity improvement obtainable whilst using the same RF power level as quadrature mode excitation, the second minimised the RF power level while maintaining approximately the same homogeneity as quadrature excitation. Figure 1 shows how these solutions were obtained using L-curves calculated for one of the study subjects. Imaging experiments were performed for the first solutions (maximised homogeneity) where measurable differences in B_1_^+^ were expected; in order to keep the examination duration within 1 h for each subject, the minimized power solutions were not used for imaging.

### Imaging experiments

RF shimming experiments were carried out on healthy volunteers, with the focus being thigh and pelvis imaging. A six-element phased array coil was used for signal reception. Research ethics committee approval was obtained for the study, and all participants gave written informed consent prior to enrolment. In total, seven healthy volunteers (two male, five female) underwent pelvic and/or thigh scanning (five volunteers underwent both).

Axial B_1_^+^ field maps were acquired using the actual flip angle imaging sequence [8] including a modified spoiling regime [9], slice profile correction [10] and linear combination mapping to improve SNR [11–13]. Imaging parameters were FOV = 440 mm × 220 mm, 64 × 32 matrix, slice thickness 10 mm, nominal flip angle = 80º and pulse repetition times of 30 ms and 150 ms. A T1-weighted turbo spin echo (T1w-TSE) sequence (440 mm × 220 mm FOV, 400 × 318 matrix, TR = 605 ms, TE = 10 ms, TSE turbo factor = 3) and a T2-weighted turbo spin echo (T2w-TSE) sequence (440 mm × 220 mm FOV, 400 × 318 matrix, TR = 2,000 ms, TE = 100 ms, TSE turbo factor = 10) were chosen for body imaging so the effect of varying the number of channels had on image quality could be assessed. All imaging was performed in the transverse plane using a single slice acquisition. The TSE sequences outlined above are often used to acquire multiple interleaved slices; use of a single slice allows the SAR to be kept as low as possible while maintaining the same contrast parameters as used in clinical examinations. A full examination, consisting of B_1_^+^ mapping, RF shim calculation and acquisition of images for all channel combinations, could be completed within 45 min. B_1_^+^ maps were also obtained for each optimized shim setting to allow comparison between the predicted and achieved shimmed B_1_^+^ distributions, independent of the receiver coil.

### RF safety and SAR analysis

Accurate calculation of SAR, particularly the local SAR, requires subject specific E-field models based on the individual’s anatomy. Obtaining this information and completing the required calculations remains a challenge to achieve during an examination and is a current research topic in its own right [14, 15]. In this study whole body SAR was dealt with simplistically using the ||**Rb**||^2^ term in the RF shimming cost function (Eq. 2). This method allowed for a time-efficient, indirect minimisation of global SAR through the limiting of total RF power. A single-subject, single-position E-field model was also used to calculate the local SAR prior to every scan performed using the method from Ref. [16], allowing for quantification of the SAR reduction potential of multiple transmit channels. The E-field model is for a large male subject, positioned with torso at isocentre, therefore the results acquired are unlikely to be accurate for different body shapes and different body positions relative to the RF coils. Due to the uncertainty in the SAR taken from this model, a maximum estimated SAR level of 10 % of the relevant regulatory limit [17] was allowed for any imaging sequence, to allow a tenfold margin for error.

### B_1_^+^ homogeneity and image quality assessment

Homogeneity was assessed for acquired B_1_^+^ maps using both root mean square error (RMSE) and coefficient of variation (*C*_V_). Both measures considered only the homogeneity of the magnitude of the measured B_1_^+^. RMSE quantifies the deviation in absolute terms from the desired field pattern, and is equivalent to the square root of the first term in Eq. 2. The coefficient of variation (standard deviation divided by mean) is purely a measure of spatial homogeneity; unlike RMSE, *C*_v_ is not altered by scaling the B_1_^+^ fields. We hypothesised that increasing the number of channels would improve homogeneity, and therefore decrease the *C*_V_. Statistical analysis was carried out on the *C*_V_ data using SPSS (IBM). Kolmogorov–Smirnov tests were used to test the normality of the data (there was no evidence for non-normality), then paired sample t-tests were used to examine differences in *C*_V_ for incremental increases in the number of channels controlled (2 vs. 1 (quad), 4 vs. 2, 8 vs. 4). The requirement for statistical significance was *p* < 0.05 for one-tailed comparisons (i.e. the alternative hypothesis is that *C*_V_ for the configuration with more channels is less).

A blind grading system was used to assess changes in imaging performance. An experienced radiologist was given all images in a randomised order and asked to grade them according to a five-point scoring system, previously described by Willinek et al. [4], but modified as all volunteers were healthy and no pathology was assessed. Perceived image quality was rated on a scale of 1–5, with 1 being an image of non-diagnostic quality and 5 demonstrating optimal contrast with no B_1_^+^ inhomogeneity artefacts.

## Results

### B_1_^+^ homogeneity and Image quality improvements

**Fig. 2:**
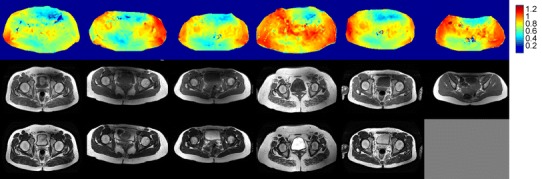
Nominal quadrature mode axial pelvis results for multiple volunteers. Axial AFI transmit sensitivity maps (S(**r**)) (*top*), T_1_w-TSE images (*middle*) and T_2_w-TSE images (*bottom*). Homogeneity is variable in all volunteers. Shading artefacts are observed particularly in the T_1_w images. Note that the T2w image is missing for one volunteer because technical problems led to the examination being terminated before all data had been acquired

**Fig. 3:**
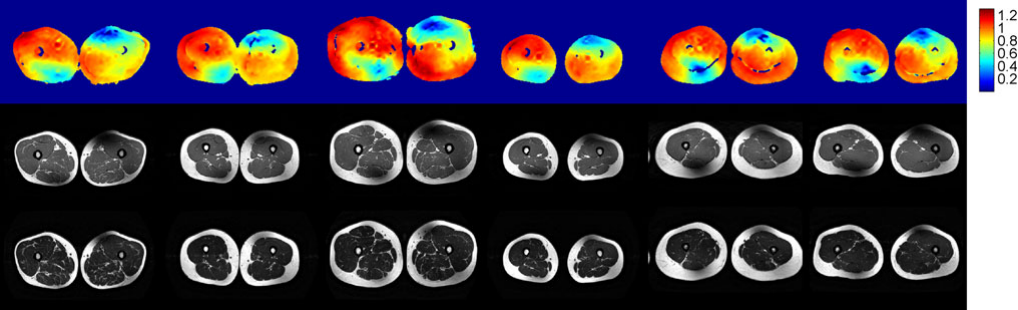
Nominal quadrature mode axial thigh results for multiple volunteers. Axial AFI transmit sensitivity maps (*top*), T_1_w-TSE images (*middle*) and T_2_w-TSE images (*bottom*). Significant areas of low sensitivity are seen in the *upper right* and *lower left* portions of the field of view with corresponding shading artefacts present on the T_1_w and T_2_w images

A consistent pattern of substantial inhomogeneity was found in the B_1_^+^ field generated by quadrature excitation, in both the pelvis and thighs of all volunteers. The inhomogeneity was characterised by diagonally offset areas of low B_1_^+^ intensity in the anterior and posterior regions. This resulted in areas of very low signal intensity in the resulting T_1_w-TSE images; the T_2_w-TSE images were more robust to the inhomogeneity. The B_1_^+^ fields and the corresponding T_1_w and T_2_w images acquired in quadrature mode for all volunteers are shown in Fig. 2 for the pelvis and Fig. 3 for the thighs. Note that the variability seen in the average B_1_^+^ value between different subjects in Fig. 2 is the result of inaccuracy in the scanner’s standard global power-optimisation procedure [18].

**Fig. 4:**
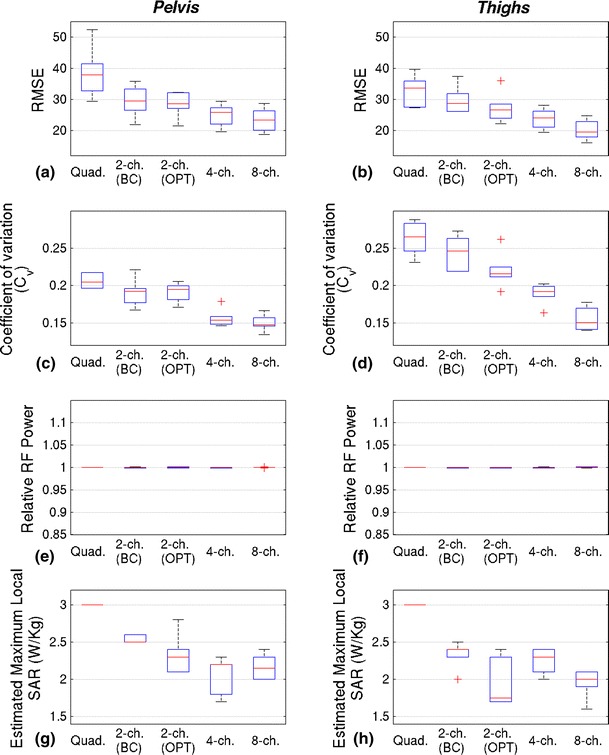
Results for experiments designed to maximise homogeneity at constant RF power. Pelvis results are shown in the *left hand column*, with thigh results in the *right column*. The maximum whisker length on all *box* plots is 1.5 × the interquartile range; all points outside this are deemed outliers and marked with a *red cross*. **a**, **b** RMSE inhomogeneity reductions achieved by increasing the number of channels. **c**, **d** C_V_ inhomogeneity versus channel configuration. Note that both RMSE (a/b) and C_V_ (c/d) were measured from acquired B_1_^+^ maps, **e**, **f** RF power was held constant with respect to quadrature mode. **g**, **h** Local SAR estimate from numerical model for AFI B_1_^+^ mapping sequence versus channel configuration

**Fig. 5:**
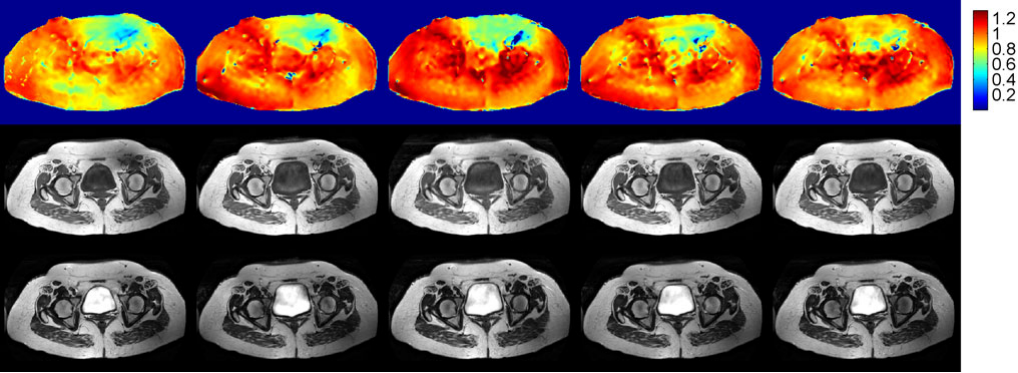
Example data from pelvis scans of one volunteer. *Top* AFI axial pelvic S(**r**) maps, *middle* T_1_w-TSE and *bottom* T_2_w-TSE axial pelvic images; columns from *left* to *right*: quadrature, 2-channel (birdcage), 2-channel (optimal), 4-channel and 8-channel. The T_2_w images appear more robust to B_1_^+^ inhomogeneity than the T1w sequence

**Fig. 6:**
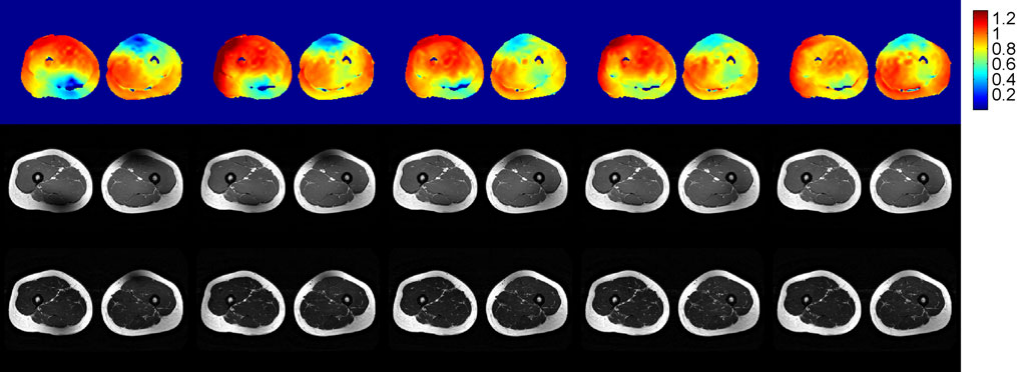
Example data from thigh scans of one volunteer. *Top* AFI axial thigh S(**r**) maps, *middle* T_1_w-TSE and *bottom* T_2_w-TSE axial pelvic images; columns from *left* to *right*: quadrature, 2-channel (birdcage), 2-channel (optimal), 4-channel and 8-channel. The T_2_w images appear more robust to B_1_^+^ inhomogeneity than the T1w sequence

Figure 4 contains RF homogeneity, power and SAR estimates for the pelvis and thigh experiments. All shim solutions had an approximately constant RF forward drive, relative to quadrature mode, as can be seen in parts e, f. The maximum local SAR as indicated by the E-field model based SAR calculator is displayed on parts g, h; note that the local SAR measurements are quoted for the AFI B_1_^+^ mapping sequence. This figure shows that by increasing the number of transmit channels, the B_1_^+^ field can be made progressively more homogeneous for both the pelvis and thighs. The results of paired sample *t*-tests comparing *C*_V_ for configurations with successively increasing numbers of channels showed that improvements were significant (Table 1) for both anatomies studied and for all increases in channel number. Examples of the effect of these improvements in single volunteers can be seen in Figs. 5 and 6, which contain the B_1_^+^ maps, T1w-TSE and T2w-TSE images obtained for each configuration from a single volunteer.

**Table 1:** Table of *p* values for pairwise comparison of *C*_V_ measured from acquired B_1_^+^ maps for configurations with increasing numbers of channels

Anatomy	*p* values		
	2-channel (opt) compared with Quadrature	4-channel compared with 2-channel (opt)	8-channel compared with 4-channel
Pelvis	0.0026	0.0005	0.0075
Thigh	0.0038	0.0020	0.0004

Taking 0.05 as the threshold for significance, increasing from one to two, two to four channels and four to eight channels yielded significant improvements in homogeneity in all cases. Note that only the optimal 2-channel configuration (not the ‘birdcage’ configuration) is shown here to reduce the number of comparisons

The mean maximum local SAR measurements from the online SAR model decreased with increasing numbers of channels for the pelvis experiment, although 4- and 8-channel configurations performed similarly. In the thighs there was also a gradual reduction with the exception of the 2-channel (non-birdcage) configuration which achieved a much lower calculated average local SAR than the other configurations. When transmitting in single-channel (nominal quadrature) mode, the maximum local SAR for the AFI sequence is always 3 W/kg according to the experimental online electric field model as no phase/amplitude modifications have been made and the system is calibrated.

The blind grading results from the anatomical images for all coil configurations averaged over all subjects are displayed in Table 2. In the case of the T1w images, increasing channel numbers also increases perceived image performance for both anatomies tested. For pelvic imaging there were no perceived improvements in the T2w images when increasing the number of transmit channels; however, a slight improvement was noted when imaging the thighs.

**Table 2:** Results of blinded image quality grading (mean ± SD across all subjects) using five point scale adapted from Willinek et al. [4]

Anatomy	Imaging sequence	Quadrature	2-channel (birdcage)	2-channel (optimal)	4-channel	8-channel
Pelvis	T1w	4.00 ± 0.63	4.33 ± 0.52	4.33 ± 0.52	4.50 ± 0.55	4.67 ± 0.52
	T2w	4.20 ± 0.45	4.60 ± 0.55	4.60 ± 0.55	4.60 ± 0.55	4.60 ± 0.55
Thigh	T1w	2.50 ± 0.55	3.20 ± 0.84	3.67 ± 1.03	4.33 ± 0.52	4.67 ± 0.52
	T2w	4.00 ± 0.63	4.60 ± 0.55	4.60 ± 0.55	4.83 ± 0.41	4.83 ± 0.41

### RF power reduction with increasing channel numbers

**Fig. 7:**
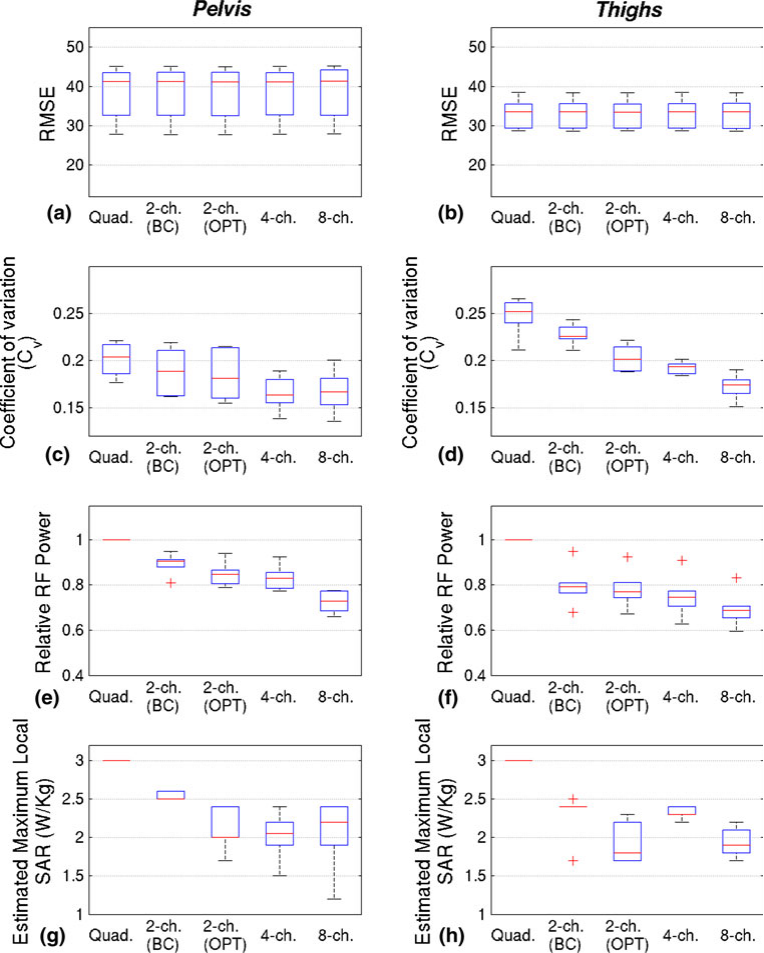
Comparisons of homogeneity, power requirements and local SAR with different numbers of channels when performing RF shimming at a fixed level of homogeneity. Unlike Fig. 4, homogeneity values (RMSE/C_V_) are predicted rather than measured. Pelvis results are shown in the *left column*, thigh results on the *right*. **a**, **b** RMSE is held the same for all configurations; variability comes from inter-subject differences. **c**, **d** C_v_ falls with increasing channel count, indicating that although the RMSE is held constant the actual field patterns do vary. **e**, **f** RF power requirements fall as the number of channels increases. **g**, **h** Local SAR prediction of AFI B_1_^+^ mapping sequence acquired with power reduction shims

For all experiments, optimized RF shims were also computed for minimizing RF power (quantified by ||**Rb**||^2^) while maintaining a fixed homogeneity level (constant RMSE). No imaging was performed for these solutions; B_1_^+^ homogeneity was instead estimated from the predicted field maps for the solutions found. The results are summarised by Fig. 7. In both anatomies, the total RF power reduces as the number of channels increases, with the 8-channel system having the lowest power requirement. The predicted local SAR is also reduced for channel counts higher than one, however these do not follow the same pattern as total RF power. In addition the C_V_ decreased with increasing channel number in both cases.

## Discussion

In this study, conducted at 3T, pronounced areas of B_1_^+^ inhomogeneity were found in the body with the areas of low B_1_^+^ intensity following a distinctive diagonal characteristic of ‘elliptic’ shaped loads, which the human body loosely resembles [19]. Our results show that increasing the number of RF channels, and hence the number of effective degrees of freedom, leads to successive improvement in the achievable B_1_^+^ homogeneity. This is visible from the diagram in Fig. 1, which was generated using one of the datasets included in the study. The solutions found for configurations with more degrees of freedom are nested within those found with fewer degrees of freedom, indicating that a more favourable trade-off between power and inhomogeneity can be achieved by increasing the channel number. A systematic study using multiple volunteers confirmed that increasing the number of channels from one to two, two to four, and four to eight all resulted in statistically significant improvements in homogeneity. Improved RF homogeneity also led to a quantifiable improvement in image quality as measured by a blinded evaluation, especially in T1 weighted images. The T2 weighted TSE sequence used was found to be more robust to B_1_^+^ homogeneity. The amplitude of a single spin echo produced by refocusing flip angle *α* is proportional to sin^2^(*α*/2), whereas the pseudo steady-state echo amplitude produced by a TSE sequence with multiple refocusing is proportional to sin(*α*/2) [20]. For both the T1 and T2 weighted sequences, *α* was 130°, however the former used centric phase encoding, while the latter used linear. As a result it would be expected that the T1 weighted images would be more influenced by the first echo (proportional to sin^2^(*α*/2)) while the T2 images would be closer to the pseudo steady-state (sin(*α*/2)), which is in accordance with the finding that the T2 weighted sequence is more robust to variations in B_1_^+^.

As well as B_1_^+^ field homogeneity, parallel transmission also allows greater control over both global and local SAR. RF shims were calculated for maximal homogeneity at a fixed RF drive power level, where drive power is the sum of squares of the RF drive levels supplied to each channel, quantified by ||**Rb**||^2^. A single station E-field model for a single subject was available for the estimation of peak local SAR for these solutions (Fig. 4g, h for pelvis and thigh respectively). These results indicate that there was some reduction in local SAR for the shimmed solutions, but importantly none of the shimmed solutions produced a higher local SAR than quadrature mode, according to the model. It should be noted that this optimisation did not aim to minimise local SAR, and in fact held relative power constant. A second set of experiments which held homogeneity constant and aimed to reduce total power did indeed show consecutive reductions in total power as channel number increased. The local SAR, as predicted by the model, also decreased when compared with quadrature mode, but again decreases were erratic. This is partly to be expected because the SAR model did not accurately describe the imaging scenarios, either in the positioning or the size and build of the subject. Personalised models for each subject might allow a subject-specific estimation of the local SAR within the current framework. These could be used to much greater effect by allowing the replacement of the regularization term in Eq. 2 with a term based directly on a true SAR estimate. A recent study of this type by Homann et al. [15] investigated shimming with an 8-channel coil of a similar design to the one used in this study. In that study, it was found that including a subject-specific SAR model when determining shim settings produced substantial SAR reductions, which is in line with our findings using a single standard model and in relation to total input power. The method employed in the current study, in which an L-curve is calculated to explore balancing B_1_^+^ homogeneity with total drive power, or ideally with peak local SAR, allows flexible choice of operating point. In this study, constant power or constant homogeneity solutions were examined, but in practice any point along the curves could be selected, depending on the situation. As indicated by the imaging examples presented in Figs. 2, 3, 5 and 6, different sequences have variable sensitivity to B_1_^+^ inhomogeneity. The T2w sequence employed here is more robust, and so a lower power solution might be selected to increase efficiency, whereas other parts of an examination may be much more vulnerable and so warrant a different homogeneity-power trade-off. This allows flexible balancing of SAR load across a whole examination. At the other extreme, Fig. 1 shows that it is possible to achieve highly homogeneous B_1_^+^ fields over a large (axial) field of view with a multi-transmit system at 3T if the total power is not a concern. However, useful solutions emerge as soon as any power considerations are introduced, in the case of the method used here, by setting λ to any non-zero value.

The lack of suitability of the SAR model for the subjects imaged meant that the true SAR values were uncertain. For this reason, a conservative approach was taken, and imaging was limited to run at 10 % of the relevant SAR limit. This limitation is the subject of a considerable research effort in the wider community: the use of generic models [21], simplified models generated in real time [22] or even actual in situ measurement of the SAR distribution [23] are all current possibilities. More accurate models would allow subject-specific SAR reductions to be calculated; if predictions could be treated with confidence, then reductions in quoted SAR would be translatable into an increase in permissible RF duty cycle, which can be used to reduce TR or increase the maximum number of slices within a fixed TR. If the number of slices were increased, extra B_1_^+^ maps would also need to be acquired, potentially slowing the acquisition process. New methods such as that in Ref. [24] provide the possibility of rapid B_1_^+^ measurement over large volumes, alleviating this issue. Similarly, while computation times were acceptable for this study, they would not scale well to multiple slices. The code used was, however, not optimized for speed since it was not an issue here, though it could be accelerated if necessary.

The human body, with its approximately elliptic cylindrical geometry, produces field variation mainly in the axial plane [19]. As a result, prevailing coil designs consist of elements distributed azimuthally around the magnet, giving maximal axial control. Consequently, in this study all imaging and field measurements were obtained in axial planes, where the effects of field inhomogeneity are generally greatest and the existing coil design offers its maximum flexibility in field control. We have found qualitatively similar performance differences between the different channel combinations for coronal orientation scanning. Given the asymmetry in the coil design, with its lack of any head-foot segmentation, the manner in which changing the orientation of the scanning plane modulates the magnitude of the effect of the number of degrees of freedom exploited is likely to be strongly influenced by the properties of the coil. A systematic investigation of these effects in the context of an array configuration that has multiple elements arrayed in all three directions around the body will be of further interest and the current work suggests that such additional degrees of freedom would result in a powerful capacity for homogeneity control. Similarly, while this study was conducted purely at 3T, B_1_^+^ inhomogeneity issues are greater at higher field strengths. The results of this work cannot directly be translated to these other scenarios, but they do indicate that transmit arrays, perhaps with more than 8 channels, could mitigate related issues at field strengths greater than 3T.

## Conclusion

The impact the number of RF transmit channels has on B_1_^+^ homogeneity, RF power requirements/SAR reduction and image quality when performing subject-specific RF shimming was investigated. Increasing the number of transmit channels was found to yield progressive improvements in B_1_^+^ homogeneity achieved under approximately constant total drive power, or reduction in total drive required to achieve the same level of B_1_^+^ homogeneity. The regularised shimming approach adopted in this study allows B_1_^+^ homogeneity and total RF power to be traded against one another and the balance adjusted throughout an examination to optimise imaging performance and control RF power deposition. RF shimming with large coil arrays allows for significant increases in homogeneity, beyond those found with 2-channels. In this study, the maximum number of channels was 8. Potentially, further performance increases could be achieved in some anatomies with a greater number of transmit channels, with the use of suitable array coils.
